# Effects of others’ gaze and facial expression on an observer’s microsaccades and their association with ADHD tendencies

**DOI:** 10.1186/s40101-023-00335-2

**Published:** 2023-09-07

**Authors:** Yuki Motomura, Sayuri Hayashi, Ryousei Kurose, Hiroki Yoshida, Takashi Okada, Shigekazu Higuchi

**Affiliations:** 1 Department of Human Life Design, Faculty of Design, Kyushu University, 4-9-1 Shiobaru, Minamiku, Fukuoka, 815-8540 Japan; 2 Department of Kansei Science, Graduate School of Integrated Frontier Science, Kyushu University, 4-9-1 Shiobaru, Minamiku, Fukuoka, 815-8540 Japan; 3 Department of Developmental Disorders, National Institute of Mental Health, National Center of Neurology and Psychiatry, 4-1-1, Ogawahigashi, Kodaira, Tokyo, 187-0031 Japan

**Keywords:** Microsaccades, Attention, Gaze, Emotion, Oculomotor control, Attention-deficit/hyperactivity disorder

## Abstract

**Background:**

The aim of this study was to examine the effect of others’ gaze on an observer’s microsaccades. We also aimed to conduct preliminary investigations on the relationship between the microsaccadic response to a gaze and a gazer’s facial expression and attention-deficit/hyperactivity disorder (ADHD) tendencies.

**Methods:**

Twenty healthy undergraduate and graduate students performed a peripheral target detection task by using unpredictable gaze cues. During the task, the participants’ eye movements, along with changes in pupil size and response times for target detection, were recorded. ADHD tendencies were determined using an ADHD questionnaire.

**Results:**

We found that consciously perceiving the gaze of another person induced the observer’s attention; moreover, microsaccades were biased in the direction opposite to the gaze. Furthermore, these microsaccade biases were differentially modulated, based on the cognitive processing of the facial expressions of the gaze. Exploratory correlation analysis indicated that microsaccade biases toward gazes with fearful expressions may specifically be correlated with participant characteristics, including inattention.

**Conclusions:**

Our findings support that microsaccades reflect spatial attention processing and social cognitive processing. Moreover, the exploratory correlation analysis results suggested the potential benefit of using microsaccade bias toward spatial attention to assess pathophysiological responses associated with ADHD tendencies.

**Supplementary Information:**

The online version contains supplementary material available at 10.1186/s40101-023-00335-2.

## Background

Very small unconscious eye movements occur when humans gaze at an object. These miniature movements have been reported as randomly occurring oculomotor phenomena and are considered fixational eye movements. Fixational eye movements are subdivided, based on their dynamic characteristics, as follows: tremors with small and high frequencies, drifts with large and slow movements, and microsaccades with saltatory characteristics similar to normal eye movements (i.e., saccades) [1, 2]. These movements are involved in counteracting retinal adaptation by randomly displacing the retinal image and refreshing the stimulus input into the retinal nerves [3].

Spatial attention and saccade movements are closely related and have partly common neural mechanisms [4–6]. Engbert et al. [7] investigated the effect of spatial attention shifts on microsaccades. They found that microsaccades orient toward spatial attention induced by spatial cues in a subsequent phase with increasing microsaccades (i.e., rebound phase), after the initial inhibition of microsaccades [7]. Their findings suggest that microsaccades, which are lower-order oculomotor phenomena for visual maintenance, may be associated with higher-order cognitive functions. Subsequent studies have demonstrated that microsaccades reflect various higher-order cognitive processes, including attention. For example, Valsecchi et al. [8] examined microsaccade activities using an oddball task and reported differences in temporal patterns in microsaccadic frequencies between oddball and standard stimuli. In particular, they reported prolonged initial inhibition of microsaccades in response to oddball stimuli requiring recognition and memory (i.e., counting the number of oddballs) [8]. Additionally, Kashihara et al. [9] reported that unpleasant stimuli inhibited microsaccade occurrence in the rebound phase; moreover, they suggested that the microsaccades are oriented to the opposite side of unpleasant stimuli during the rebound phase.

The microsaccade direction in the rebound phase is influenced by the spatial attention direction. Of note, microsaccade bias is dependent on the type of attention [7, 10–12], especially on the mode (i.e., endogenous or exogenous) [13]. If an arrow indicating the target position on either side is shown and spatial attention is intentionally moved to the arrow-indicated direction, the mode is endogenous attention. However, if a flash suddenly appears around the visual field and reflexive attention is given, the mode is exogenous attention. Endogenous attention is a top-down process, based on the internal state and conscious expectations. By contrast, exogenous attention is a bottom-up process reflexively controlled by prominent external sensory events [14]. The microsaccade direction is biased toward the direction of endogenous attention. The microsaccade direction after presenting stimuli that provide spatial cues is biased toward the endogenous attention shift [7]. The sudden appearance of peripheral stimuli that attract exogenous attention conversely causes microsaccade biases in the direction opposite to that of the attention shift [15, 16].

Two main interpretations exist regarding the mechanisms underlying microsaccade bias in the direction opposite to that of exogenous attention. One interpretation is that microsaccade bias reflects an attention shift due to “inhibition of return” (“IOR”) [17, 18], a phenomenon in which attention is inhibited from “returning” to the position where the attention was previously directed [19]. This facilitates attention to be provided to a position in the visual field without previous attention [19]. For example, in a task where the preceding peripheral stimuli and subsequent target stimuli are spatially matched, IOR is presumed to have occurred when a delayed reaction occurs in the previous attention direction. Another interpretation is the “inhibition hypothesis” where in case maintaining fixation is necessary, inhibiting automatic saccades induced by exogenous stimuli causes microsaccade directional bias [20]. This hypothesis is based on the finding that inhibiting reflex saccades causes bias in oculomotor neuron activation to movements into the contralateral visual field [21] in processing for integrating information of the saccade direction in the superior colliculus [22]. However, these hypotheses regarding the opposite microsaccade bias due to exogenous attention remain unclear.

When observing another person’s gaze, attention is guided in the direction of that gaze. This automatic attention orientation involves subcortical oculomotor systems, including the superior colliculus, which controls eye movements such as saccades [23]. Studies have demmonstrated that others’ eyes affect an observer’s attention and eye movements [23–25]. In a saccade task using gaze cues, the required reaction times and saccade accuracy are enhanced when the others’ gaze direction matches the target position [25, 26]. By contrast, inconsistent gaze directions have an interference effect on task performance [27]. Whether this automatic attention orientation induced by eye gaze reflects endogenous or exogenous attention remains under discussion. Previous studies have demonstrated that nonsocial spatial cues (e.g., arrows) trigger endogenous spatial attention [9, 28]. Findings of another review study [29] indicate that social-spatial cues (e.g., eye gaze) also automatically guide spatial attention; however, this attentional processing may differ from endogenous attention. Moreover, a recent study [30] showed that attention orientation induced by eye gaze resembled exogenous attention rather than endogenous attention, which suggested an association of attentional processing triggered by eye gaze with exogenous attention.

The automatic saccade control mechanism based on another person’s gaze is considered crucial for quickly detecting others’ interests and potential dangers. Saccades started and stopped by social cues (other person’s gaze) are more resistant to inhibition by interference stimuli, compared to those started and stopped by nonsocial cues (e.g., color changes) [31]. This finding suggests the biological importance of the other person’s gaze.

Thus, neural networks associated with such attention and saccade control may have neural structures specialized in processing social signals, especially information obtained from others’ gaze [32]. Furthermore, microsaccades controlled by the superior colliculus, including saccades, can be modulated by spatial attention generated by perceiving others’ gaze direction and may reflect the social value of gaze information. Exogenous attention caused by emotional stimuli and prominent visual stimuli that suddenly appear in the peripheral vision modulate the microsaccade direction and microsaccadic rates [9, 15, 16]. However, it remains unclear whether stimuli with left or right gaze and emotional facial expressions modulate these microsaccade activities. Others’ gaze contains crucial information for survival and is thought to be modulated by others’ emotions. For example, another person’s fearful gaze may act as a signal of a potential threat ahead, and the ability to perceive this gaze may have adaptive significance. One of our study objectives was to investigate how microsaccades, which are involuntary eye movements related to attentional functions, are affected by neutral or fearful gaze. Moreover, microsaccades, which are associated with both attentional and emotional processing, may be beneficial for the assessment of pathophysiological responses associated with problems in social and attentional function [9].

Attention-deficit/hyperactivity disorder (ADHD) is one of the most common neurodevelopmental disorders. ADHD is characterized by inattention and/or hyperactivity-impulsivity persistent from childhood [33]. The estimated prevalence of ADHD in adults is approximately 2.5% [34]. Compared to typically developed adults, some studies have suggested that adults with ADHD have a higher risk of facing social problems such as divorce [35], substance abuse [36, 37], driving accidents [38, 39], sexual problems [34, 40], and job changes [35]. These risks in adults with ADHD may be associated with a complex interplay of multiple factors, including cognitive deficits (i.e., impaired executive function [41] and reward systems [42]), developmental history, and social and medical support.

In addition, some studies have reported deficits in social cognitive processing in ADHD [43–45] with abnormality in oculomotor control [46]. One study [47] also reported that adults with high ADHD tendencies made more errors in a saccade task requiring oculomotor inhibitory control (i.e., antisaccade) and cognitive processing of social cues (i.e., others’ facial expressions) than did adults in the control group. Another study [48] demonstrated that attention loads are inversely correlated with the microsaccadic rate, which supports that the microsaccadic rate (i.e., the number of microsaccades per second) reflects the cognitive/attention processing level [12, 48]. Moreover, ADHD studies have reported a lower microsaccade inhibition rate in patients with ADHD than in individuals with typical development, which suggests that microsaccades reflect deficits in attentional function in patients with ADHD [49, 50]. Thus, oculomotor control, including microsaccades strongly associated with saccades [51], possibly characterizes social and attentional function, which may be useful for developing biological assessment for social and attentional dysfunction in individuals with high ADHD tendencies.

As previously mentioned, numerous studies have investigated microsaccades for spatial attention; however, few studies have investigated the effects of perception/attention on microsaccades resulting from social factors. Additionally, microsaccades may reflect an individual’s attentional function and different modulations may be prominent, especially in spatial tasks requiring cognitive processing of social cues. Therefore, this study aimed to examine whether differences in facial expressions affect the microsaccadic rates, and whether others’ gaze modulates microsaccade direction. Additionally, we aimed to examine whether microsaccade biases differ, depending on the gazers’ facial expressions. Based on previous findings indicating that negative emotional pictures suppressed the microsaccadic rate during the rebound period [9, 28], we expected that a face with negative expression would induce a lower microsaccadic rate than would a neutral face. Additionally, based on a recent study [30] demonstrating that attention orientation induced by social cues resembled that induced by reflexive exogenous cues, we expected that the microsaccade direction would be biased for opposite to gaze directions. We also expected that this directional bias would be enhanced by fearful expression (implying fear located toward the gaze) because the direction of the microsaccadic response is biased opposite to fear [9, 28]. For preliminary and exploratory examinations of the utility of microsaccades as a biomarker of ADHD tendencies, we aimed to investigate the relationship between the microsaccadic response to the gaze and the gazer’s facial expression and personal characteristics, determined by using an ADHD questionnaire.

## Methods

### Participants

We enrolled 20 healthy male university students [mean age (standard deviation [SD]): 22.90 (0.91) years old] from Kyushu University (Fukuoka, Japan). We recruited male students on account that sex possibly affects attentional processing for eye gazes [52] and emotional signals [53], and difficulties in conducting experiments using female individuals (e.g., obtaining information about menstrual cycles or changing participants’ clothes for the experiments). After receiving explanations regarding the experiment’s purpose, the participants provided written consent. This study was approved by the Research Ethics Committee of Kyushu University.

### Stimulus presentation

To present the stimuli, we selected images of six models (three male models and three female models) with neutral and fearful facial expressions from the standard facial expression stimuli set [54]. To control gaze direction, the irises and pupils of the original facial image were moved to the right or left using Adobe Photoshop CC 2018. The background and model hair were removed after cropping the images into oval shapes with a width of 3.3° and a height of 4.4°. Stimulus presentation and behavioral data acquisition (i.e., response times) were controlled using software (Presentation; Neurobehavioral Systems, Berkeley, CA, USA) on a personal computer.

### Procedure

**Fig. 1:**
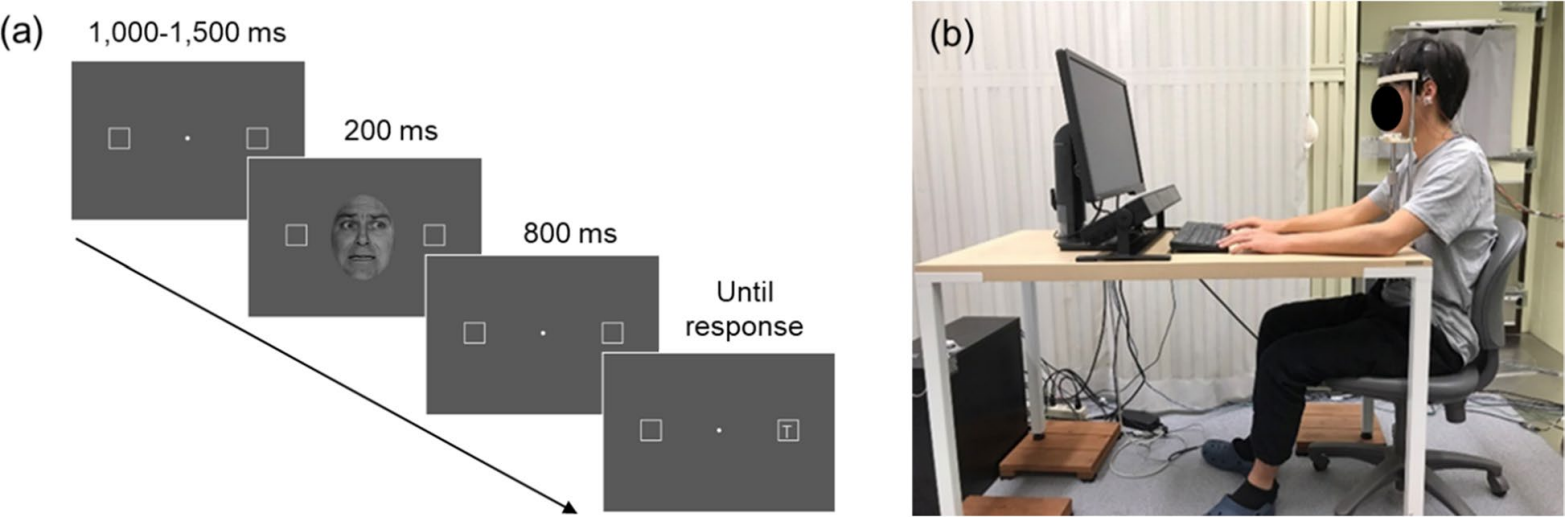
Example of a stimulus presentation sequence and experiment scene

After providing informed consent, the participants change their clothes for the experiments. Then, the participants completed a questionnaire regarding their personality traits and drowsiness. They subsequently sat in front of a display with the height of the chin rest adjusted to achieve a comfortable position. After calibrating the measuring instrument, a peripheral target detection task was conducted using the procedure shown in Fig. 1 (a).

The stimulus presentation was started with a white gazing point (・, viewing angle: 0.19°) and two boxes (□, size: 2° × 2°; line width: 0.35°; distance from the gaze point: 8°) on a gray background presented for 1000–1500 ms, as shown in Fig. 1 (a). The face images (neutral or fearful expression, left or right gaze) randomly selected from the dataset were then presented in the display center for 200 ms. After the facial image disappeared, the gaze point subsequently reappeared in the center for 800 ms; the target stimuli (“T”, size: 0.7° × 0.7°) were presented at the left or the right box. The participants were instructed to promptly report the target stimulus location (i.e., left or right) by pressing the left and right control keys on the keyboard with their index fingers. The target stimulus was displayed until the response. The task interval was set at 4 s.

The experiment comprised six blocks. Each block consists of 48 trials. Within each block, each facial stimulus (a total of 24 stimuli: two facial expressions × two gaze directions × six models) was presented two times. The position of the target stimuli was presented, regardless of the gaze direction of the previously presented face image (50% matches the gaze direction). Additionally, the program was controlled to ensure the same number of matching and mismatching trials in each block. Moreover, the participants were informed that the gaze point of a presented face would be irrelevant to the target direction. Further, they were instructed to always adjust the viewpoint to the gaze and to avoid extensively blinking during the stimulus presentation.

### Experiment environment

The experiment was conducted in a soundproof/electromagnetic-shielded artificial climate room (set temperature: 25 °C; relative humidity: 60%) in the Environmental Adaptation Laboratory, Ohashi Campus, Kyushu University [Fig. 1 (b)]. The laboratory was equipped with a web camera for observation, a 24-inch liquid crystal display (BENQ XL2411P; BenQ Corp.; Taipei, Taiwan) for presenting images, an electroencephalographer, a keyboard for responses, and an eye-movement-measuring instrument (Tobii Pro Spectrum; Tobii Technology KK, Stockholm, Sweden). The presentation display was positioned so that the distance between the participants and the gaze point was 75 cm. Additionally, the keyboard position was adjusted for each participant to allow them to press keys while seated with a comfortable posture.

### Measurement

#### Measurement of eye movements

Eye movements with a change in pupil size were tracked using the eye-movement-measuring instrument (Tobii Pro Spectrum, Tobii Technology KK; sampling rate, 600 Hz; accuracy, 0.3°; precision, 0.06°) and software (Tobii Pro Lab Version 1.98; Tobii Technology KK). A standard five-point calibration was conducted at the start of the experiment.

#### Conner’s adult ADHD rating scale–Japanese version

The ADHD characteristics of the individuals were examined using the Japanese version of Conners’ Adult ADHD Rating Scale (CAARS) [55]. The Japanese version of CAARS evaluates 66 question items related to respondents’ behaviors and problems on a four-point scale. This questionnaire yields an inconsistency index that evaluates the validity of answers to the eight subscales for ADHD, including four factor-derived subscales, three Diagnostic and Statistical Manual of Mental Disorders, Fourth Edition (DSM-IV) ADHD symptom subscales, and an ADHD index. The four factor-derived subscales are used to assess symptoms and behavior (i.e., inattention/memory problems, hyperactivity/restlessness, impulsivity/emotional lability, and problems with self-concept) across multiple domains. Moreover, the ADHD symptom subscales of the DSM-IV is used to evaluate ADHD symptoms (i.e., inattentive, hyperactive/impulsive, and total ADHD symptoms) based on the DSM-IV criteria [56]. These ADHD indicators allow differentiating between individuals with and without ADHD.

The raw scores of these subscales were converted to standard T scores on the corresponding profile paper and included for the analysis. The T scores are common to all subscales and are used as the standard scores when the mean value and SD of a large standard base (*N* = 2000) are 50 and 10, respectively. The CAARS guidelines provide percentile values corresponding to T scores for determining the status of the examined person.

In this study, an inconsistency index score of ≥ 8 points indicated an unnatural contradiction in the answer, which was then excluded from the analysis, based on the guidelines.

### Analysis method

Emotional stimuli changed the observer’s pupil diameter, based on their alertness level, which is attributed to emotional alertness adjusting pupil diameter through sympathetic nervous system activity [57]. Therefore, we analyzed pupil diameters to evaluate the alertness level by stimuli of the presented emotional facial expressions. The time-series data of the pupil diameters were epoched in the interval from -200 ms to 1000 ms relative to the onset of facial stimulus presentation. Furthermore, for baseline correction, the value of each sampling point was divided by the mean value obtained within the -200 ms to 0 ms interval of each epoch.

With regard to the analysis of eye movements, we analyzed the interval from -200 ms to 1000 ms relative to the onset of a facial stimulus presentation from the tracking data. By using a fixation filter attached to the tracking analysis software [58], intervals where blinking or saccades occurred, as well as 100 ms before and after the interval, were set as missing values. Moreover, we calculated the average eye angular velocity of 10 ms (i.e., a 20-ms time window) before and after each sample point. An interval of ≥ 30°/s was labeled as the interval when saccades occurred [58]. We excluded trials in which the defective intervals exceeded 25% of the total in each task. For the cleaned data, microsaccades were detected using a binocular microsaccade detection algorithm [7]. Microsaccades are defined as ballistic binocular movements [59]. These feature values are within the range reported by Martinez et al. [60]; therefore, the detected microsaccades were considered valid (see Supplementary file).

We excluded two participants: one participant had technical problems in the eye movement measurement and extremely few trials for analysis, whereas the other participant had undetectable microsaccades. Finally, we included 18 individuals [mean (standard deviation [SD]) age: 22.83 (0.92) years old] in the analysis.

The microsaccade direction, peak velocity, and amplitude in each task were calculated. Furthermore, we compared the microsaccade rates and direction bias under each facial expression condition.

Microsaccade rates were calculated as the microsaccade occurrence frequency with a 200 ms time window (i.e., 100 ms before and after) with a sampling interval of 1.7 ms (1/600 s). Polar plots were created to detect the microsaccade directional bias. In addition to the rebound phase (i.e., 400–600 ms relative to the onset of facial stimulus presentation) [9], we analyzed the phase following the rebound phase (800–1000 ms relative to the onset of facial stimulus presentation) because attentional processing induced by emotional signals could persist after the rebound phase [61]. The polar plots were calculated from 12 evenly spaced directional pins (every 30°) in which the angle of the horizontal axis across the center of the screen was 0° (i.e., 180°). We set areas of interest (AOIs) as 120° to 240° and -60° to 60°. An AOI on the same side of the gaze directions of a facial stimulus was set as “same.” The other AOI on the opposite side of the gaze directions was set as “opposite.” The microsaccade occurrence rate was the number of microsaccades in each AOI (i.e., same or opposite) divided by the total number of microsaccades. We used this microsaccade occurrence rate as an index of the microsaccade direction. Additionally, we calculated the microsaccade bias scores. The microsaccade bias score was the difference between the microsaccade occurrence rates in two AOIs (i.e., same minus opposite). Positive and negative values for the microsaccade bias scores indicated bias in the same and opposite directions as the gaze, respectively. To explore ADHD factors associated with these microsaccadic responses, we analyzed the correlation of the microsaccade rates and bias in each facial expression with T scores for the eight ADHD subscales in the Japanese version of CAARS.

### Statistics

We calculated the average values for participants in all trials of each condition. We subsequently calculated the total average for all participants. All data are expressed as the mean (SD). Statistical significance was set at *p* < 0.05; moreover, *p* < 0.1 was taken as marginally significant. Statistical analyses were performed using R, version 3.5.2, statistical software (R Foundation for Statistical Computing, Vienna, Austria) [62].

In the test for reaction time, we used repeated-measures two-way analysis of variance (ANOVA) (i.e., gaze-target congruence [congruent, incongruent] × facial expression [neutral, fear]). In the test for pupil diameter changes and microsaccadic rates, we calculated the average pupil diameter and microsaccadic rate for each interval. We subsequently conducted a paired *t*-test to analyze differences between the mean values of the facial expressions.

Regarding the microsaccade directional bias, we conducted repeated-measures two-way ANOVA (i.e., microsaccade direction [same, opposite] × facial expression [neutral, fear]) for the microsaccade rate in each interval. When an interaction existed, a simple main effect analysis with Bonferroni’s correction was conducted.

As the exploratory correlation analysis for the preliminary investigation on the relationships between microsaccadic responses and each scale of the Japanese version of the CAARS, we calculated Pearson’s product-moment correlation coefficient with testing of the significance of the correlation coefficient without multiple comparison corrections.

## Results

### Reaction time

Table 1 shows the mean reaction time (RT) for each experimental condition. A two-way ANOVA with the gaze-target congruence and facial expression as factors revealed a significant main effect of gaze-target congruence [*F*(1,17) = 8.58, *p* = 0.01, partial *η*^2^ = 0.34]. However, the main effects of facial expressions [*F*(1,17) = 2.61, *p* = 0.12, partial *η*^2^ = 0.] and interactions [*F*(1,17) = 1.59, *p* = 0.22, partial *η*^2^ = 0.09] were not significant.

**Table 1:** Average reaction time under each condition

	**Neutral**		**Fear**	
**Congruence**	**Congruent**	**Incongruent**	**Congruent**	**Incongruent**
**Mean RT (ms)**	306.60	310.80	302.62	310.73
**SD**	32.78	37.02	32.99	33.00

*RT* reaction time, *SD* standard deviation

### Pupil diameter

**Fig. 2:**
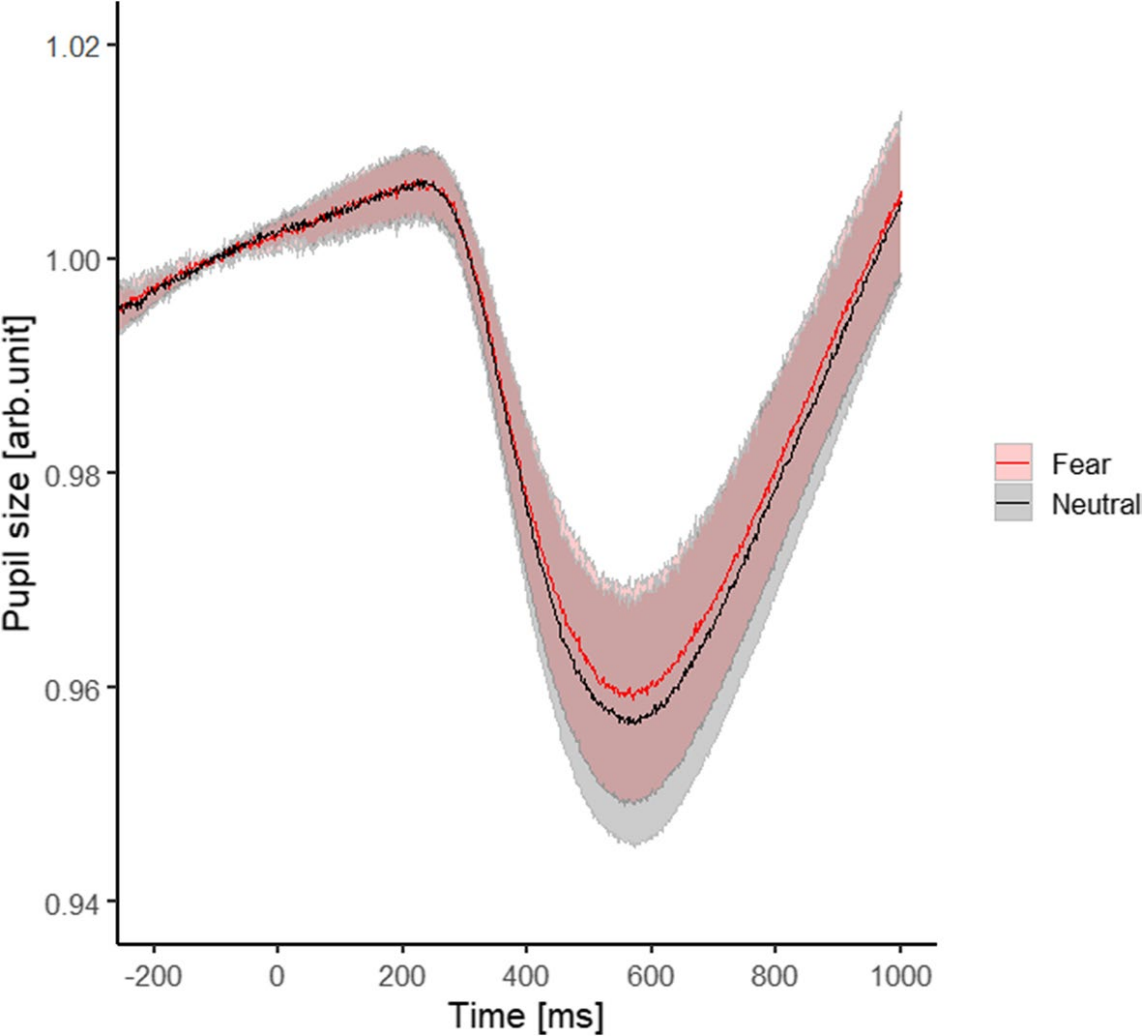
Time-series change in pupil size for each facial expression

Figure 2 shows the time-series changes in the pupil diameter. For each facial expression, the pupil diameter began decreasing at approximately 300 ms relative to the onset of facial stimulus presentation, reached a minimum at approximately 600 ms, and then returned to baseline.

To confirm the effect of facial expressions on pupil diameter in the interval showing inhibition (i.e., 300–1000 ms relative to the onset of facial stimulus presentation), we conducted a paired *t*-test by using the mean value of the pupil diameter of each facial expression within this interval. However, we observed no effect resulting from differences in facial expressions [*t*(17) = 0.51, *p* = 0.62, Hedges’ g = 0.03].

### Microsaccade rates

**Fig. 3:**
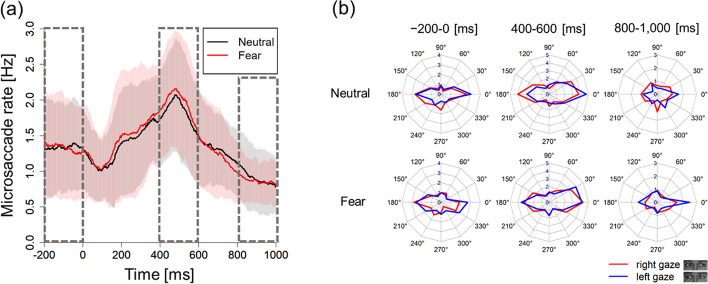
Time-series change in the microsaccadic rates and polar plots in microsaccade directions in each phase for each facial expression. Boxes indicated by the dotted line in (a) show three intervals for further analysis

Figure 3 (a) shows the time-series change in the microsaccade rates. The microsaccadic rates relative to the onset of a facial stimulus presentation (i.e., 0–200 ms) decreased under each condition (i.e., neutral and fear) and subsequently increased beyond the baseline (i.e., 400–600 ms, the rebound phase).

To verify the effect of facial expressions on the microsaccadic rates in each interval (i.e., -200 to 0 ms, 400 to 600 ms, and 800 to 1000 ms), we conducted a paired t-test on the mean microsaccadic rates for each facial expression. However, no effect of facial expression differences existed among the intervals from -200 ms to 0 ms [*t*(17) = -0.13, *p* = 0.90, Hedges’ g = -0.01], 400–600 ms [*t*(17) = 1.19, *p* = 0.25, Hedges’ g = 0.14], and 800–1000 ms [*t*(17) = -0.63, *p* = 0.54, Hedges’ g = -0.11].

### Microsaccade directional bias (Microsaccade occurrence rates in the same and opposite directions of gaze)

Figure 3 (b) presents the polar plots showing the microsaccade directions in which each facial expression occurred within the target intervals (-200 to 0 ms, 400–600 ms, and 800–1000 ms). In the interval from -200 ms to 0 ms relative to the onset of facial stimulus presentation, which was the baseline, no main effect existed for microsaccade direction [*F*(1,17) = 2.56, *p* = 0.13, partial *η*^2^ = 0.13] or facial expression [*F*(1,17) = 0.54, *p* = 0.47, partial *η*^2^ = 0.03], and no interaction effect existed [*F*(1,17) = 0.01, *p* = 0.92, partial *η*^2^ < 0.001].

**Fig. 4:**
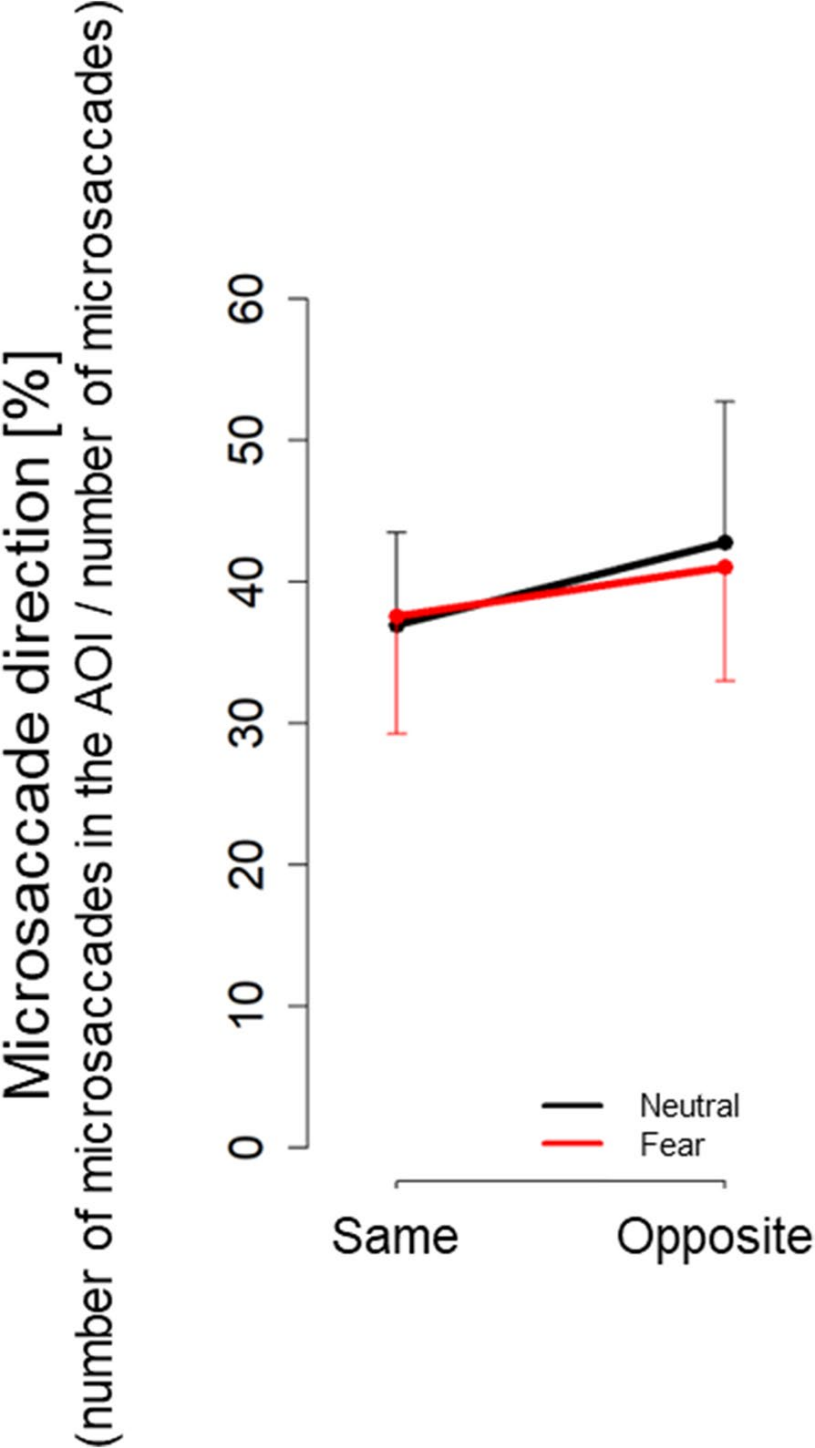
Microsaccade direction between 400 and 600 ms relative to the onset of facial stimulus presentation under each condition

By contrast, in the interval from 400 to 600 ms relative to the onset of a facial stimulus presentation, the microsaccade direction had a main effect [*F*(1,17) = 7.49, *p* = 0.01, partial *η*^2^ = 0.31; Fig. 4]. However, no significant main effect existed for facial expressions [*F*(1,17) = 0.42, *p* = 0.52, partial *η*^2^ = 0.02] and interaction effect [*F*(1,17) = 0.23, *p* = 0.64, partial *η*^2^ = 0.01]. This finding indicated that the observer’s microsaccades were biased in the direction opposite to the gaze, regardless of differences in facial expressions.

**Fig. 5:**
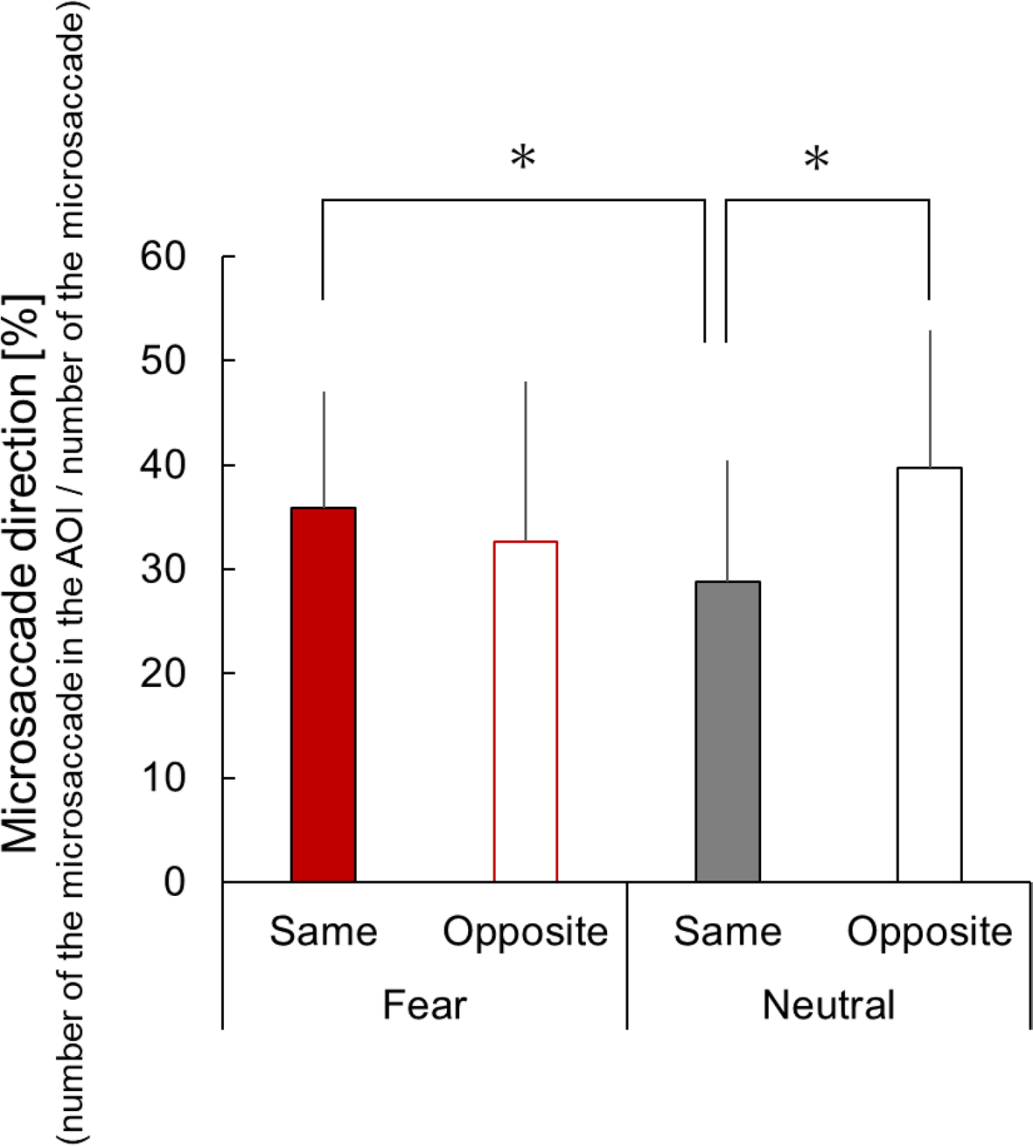
Microsaccade direction between 800 and 1000 ms relative to the onset of facial stimulus presentation under each condition

In the interval from 800 to 1000 ms relative to the onset of facial stimulus presentation, no significant main effects existed for the microsaccade direction [*F*(1,17) = 1.07, *p* = 0.32, partial *η*^2^ = 0.06] and facial expression [*F*(1,17) = 0.0008, *p* = 0.98, partial *η*^2^ = 0.001]. However, a significant interaction effect existed between the microsaccade direction and facial expression [*F*(1,17) = 4.54, *p* = 0.05, partial *η*^2^ = 0.21]. Simple main effect analysis revealed a higher proportion of microsaccades in the same direction as the gaze induced by fear than neutral [*F*(1,17) = 7.76, *p* = 0.01, partial *η*^2^ = 0.31; Fig. 5 (a)]. A simple main effect analysis revealed that the effects of a direction is significant only in the neutral condition [neutral: *F*(1,17) = 4.59, *p* = 0.05, partial *η*^2^ = 0.21; fear:* F*(1,17) = 0.44, *p* = 0.15, partial *η*^2^ = 0.12]. The mean (SD) microsaccade bias scores in the neutral and fear conditions were -10.9 (21.6) and 3.24 (20.6), respectively. These findings showed that the microsaccade direction in the interval of 800 ms to 1000 ms was biased opposite to the gaze in the neutral condition.

### Correlation with questionnaire CAARS

**Fig. 6:**
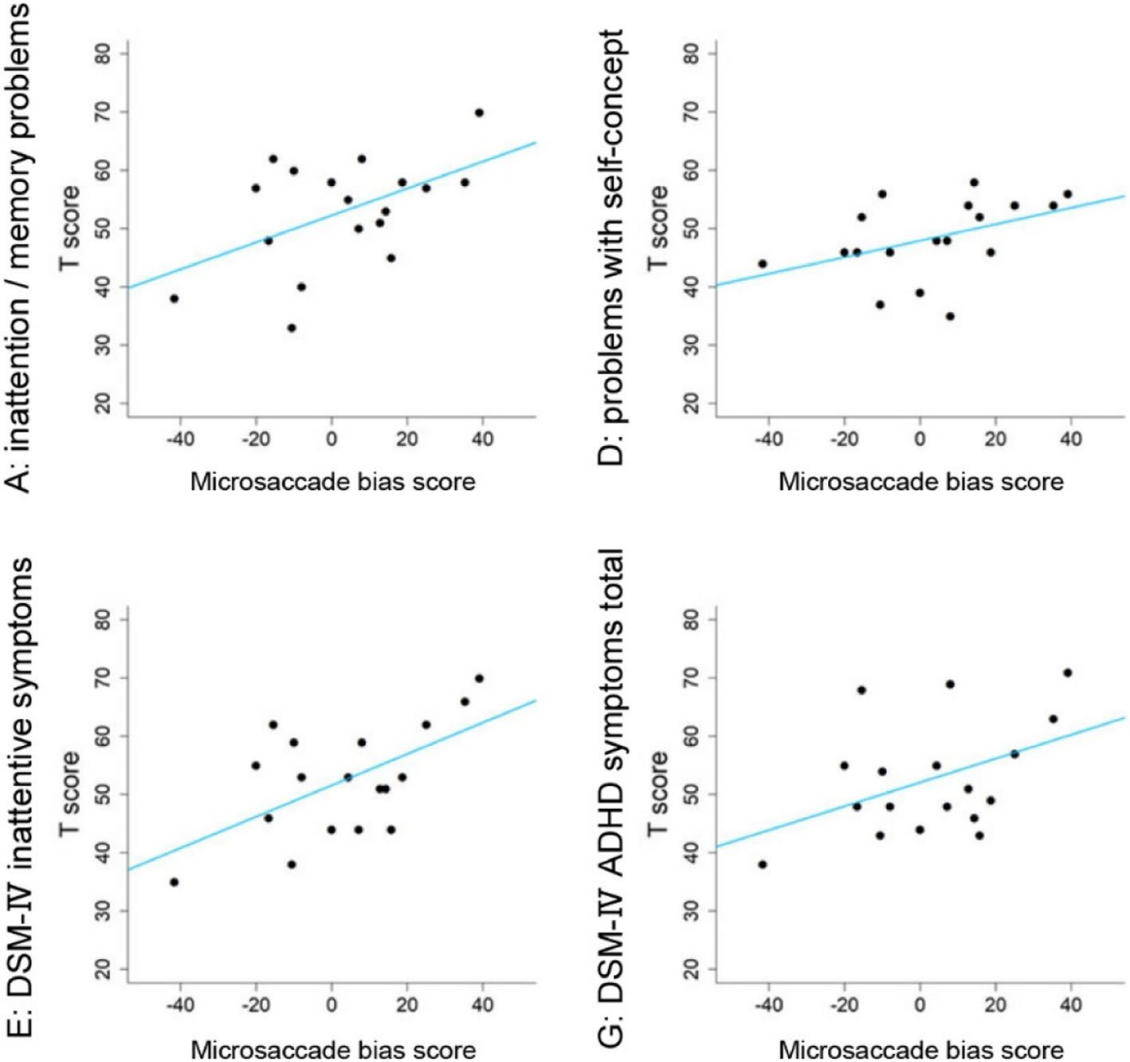
Correlations with microsaccade biases induced by fearful expressions from 800 to 1000 ms relative to facial stimulus onset

Table 2 shows descriptive statistics of the CAARS T scores. Table 3 shows the results of the correlation analysis of the microsaccadic rates for each facial expression (400–600 ms and 800–1000 ms, relative to the onset of facial stimulus presentation) and microsaccade directional bias (400–600 ms and 800–1000 ms, relative to the onset of facial stimulus presentation) with the T scores of the eight subscales of the Japanese version of CAARS (i.e., inattention/memory problems, hyperactivity/restlessness, impulsivity/emotional lability, problems with self-concept, DSM-IV inattention problems, DSM-IV hyperactive/impulsive symptoms, DSM-IV total ADHD symptoms, ADHD Index). As shown in Table 3, correlations existed in the microsaccade rate and microsaccade directional bias score in the interval of 800 ms to 1000 ms relative to the onset of presenting fearful expressions with inattention/memory problems (rate: *r* = -0.51, *p* = 0.03; bias score: *r* = 0.51; *p* = 0.03), which indicated relationships between low microsaccadic responses (low rate and attenuated bias opposite to the gaze) in the interval and high-score of the inattention/memory problems. Moderate positive correlations of the microsaccade directional bias also existed in the interval from 800 to 1000 ms relative to the onset of presenting fearful expressions with the problems with self-concept (*r* = 0.43, *p* = 0.07), DSM-IV inattentive symptoms (*r* = 0.58, *p* < 0.001), and DSM-IV total ADHD symptoms (*r* = 0.44, *p* = 0.07) (Fig. 6). In the interval from 400 to 600 ms, microsaccade directional bias was moderately positive in correlation with the problems with self-concept.

**Table 2:** Summary of the CAARS T-score

	**CAARS subscale (T-score)**							
	**A**	**B**	**C**	**D**	**E**	**F**	**G**	**H**
Mean (SD)	53.1 (9.4)	53.0 (8.5)	46.9 (6.8)	48.4 (6.7)	52.5 (6.7)	52.6 (9.6)	52.8 (9.6)	51.3 (9.0)
Min–Max	33–70	42–77	40–63	35–58	35–70	41–75	38–72	34–73

The letters A to H indicate the subscale of the CAARS, as follows: A, inattention/memory problems; B, hyperactivity/restlessness; C, impulsivity/emotional lability; D, problems with self-concept; E, DSM-IV inattentive symptoms; F, DSM-IV hyperactive-impulsive symptoms; G, DSM-IV ADHD symptoms total; and H, ADHD index

*CAARS* Conners’ Adult ADHD Rating Scale, *SD* standard deviation, *Min* minimum, *Max* minimum

**Table 3:** Summary of correlation between microsaccade responses and CAARS

		A	B	C	D	E	F	G	H
Microsaccade rate (400–600 ms)	Neutral	*r* = 0.30, *p* = 0.22	*r* = 0.35, *p* = 0.15	*r* = 0.10, *p* = 0.69	*r* = 0.11, *p* = 0.67	*r* = 0.20, *p* = 0.42	*r* = 0.25, *p* = 0.31	*r* = 0.27, p = 0.27	*r* = 0.27, p = 0.27
	Fear	*r* = 0.13, *p* = 0.61	*r* = 0.09, *p* = 0.72	*r* = -0.14, *p* = 0.58	*r* = 0.13, ***p* = 0.06**	*r* = 0.02, *p* = 0.94	*r* = 0.08, *p* = 0.75	*r* = 0.07, p = 0.77	*r* = 0.18, p = 0.47
Microsaccade bias (400–600 ms)	Neutral	*r* = 0.38, *p* = 0.12	*r* = 0.33, *p* = 0.18	*r* = 0.36, *p* = 0.14	*r* = 0.04, *p* = 0.87	*r* = 0.34, *p* = 0.17	*r* = 0.20, *p* = 0.43	*r* = 0.29, p = 0.23	*r* = 0.32, p = 0.20
	Fear	*r* = 0.06, *p* = 0.82	*r* = 0.21, *p* = 0.41	*r* = 0.24, *p* = 0.33	*r* = 0.05, *p* = 0.84	*r* = 0.02, *p* = 0.94	*r* = 0.10, *p* = 0.69	*r* = 0.07, p = 0.78	*r* = 0.03, p = 0.91
Microsaccade rate (800–1000 ms)	Neutral	*r* = -0.18, *p* = 0.47	*r* = 0.08, *p* = 0.75	*r* = -0.19, *p* = 0.44	*r* = -0.18, *p* = 0.47	*r* = -0.24, *p* = 0.33	*r* = 0.04, *p* = 0.88	*r* = -0.10, p = 0.70	*r* = -0.004, p = 0.99
	Fear	***r* = -0.51, *p* = 0.03**	*r* = -0.26, *p* = 0.92	*r* = -0.24, *p* = 0.33	*r* = -0.30, *p* = 0.23	*r* = -0.34, *p* = 0.16	*r* = -0.08, *p* = 0.74	*r* = -0.23, p = 0.35	*r* = -0.22, p = 0.38
Microsaccade bias (800–1000 ms)	Neutral	*r* = 0.08, *p* = 0.75	*r* = 0.16, *p* = 0.52	*r* < 0.001, *p* = 0.99	*r* = -0.17, *p* = 0.51	*r* = -0.11, *p* = 0.65	*r* = 0.06, *p* = 0.81	*r* = -0.02, p = 0.93	*r* = -0.2, p = 0.42
	Fear	***r* = 0.51, *p* = 0.03**	*r* = 0.15, *p* = 0.55	*r* = 0.17, *p* = 0.50	***r* = 0.43, *p* = 0.07**	***r* = 0.58, *p* = 0.01**	*r* = 0.19, *p* = 0.44	***r* = 0.44, p = 0.07**	*r* = 0.27, p = 0.28

The values of *p* < 0.05 and *p* < 0.10 are shown in red-bold and black bold, respectively. Letters A to H indicate the subscale of the CAARS, as follows: A, inattention/memory problems; B, hyperactivity/restlessness; C, impulsivity/emotional lability; D, problems with self-concept; E, DSM-IV inattentive symptoms; F, DSM-IV hyperactive-impulsive symptoms; G, DSM-IV ADHD symptoms total; and H, ADHD index

*CAARS* Conners’ Adult ADHD Rating Scale

## Discussion

This study utilized a peripheral target detection task to evaluate spatial attention extrinsically caused by gaze and facial expressions. Others’ gaze is known to automatically guide the observer’s attention, even if the subsequent target position is nonpredictive [63–66]. In this study, during the detection task, the effect of congruence between the gaze and target direction was shown in the RT. In particular, the RT was faster when the gaze direction and target position did not match than when they did match. This findings indicates that the gaze of the facial stimuli used in this study automatically guided the participants’ spatial attention.

Previous studies [67–72] have reported that fearful facial expressions enhance attention in the eye gaze direction; however, we did not observe a significant effect of facial expression or facial-gaze interaction. This findings could be attributed to the difference in stimulus-onset asynchrony (SOA) between facial and target stimuli. In previous studies [68–72], most SOAs of the reaction tasks were < 500 ms; By contrast, the SOA in our study was 1000 ms, which was approximately twice as long because of the protocol setting for the microsaccade measurement. The onset of joint attention has previously been reported to begin within 200 ms relative to the onset of the stimulus presentation; thus, the SOA was longer in our study.

Time-series changes in the microsaccadic rates to facial expressions [Fig. 3 (a)], comprised early inhibition (0–200 ms), followed by the rebound phase (400–600 ms). This microsaccade rebound is primarily suppressed during tasks involving covert attention [7, 10, 17]. Previous studies [8, 12] have reported microsaccade rebound inhibition in visual and auditory oddball tasks. By contrast, attention tasks using simple visual stimuli such as arrows, light, and color, induce nonsignificant or low rebound inhibition [7, 15]. Regarding microsaccades during the rebound period, Kashihara et al. [9] reported that emotional stimuli with dilated pupil diameter, especially those related to negative emotions, had a strong inhibitory effect. Given that emotional attention can activate early brain processing, which is the subcortical pathway [73], Kashihara et al. [9] proposed that the suppressed microsaccade rebound process for emotional stimuli was caused by retaining previously important information (e.g., threat information) at the expense of the general update process with new visual inputs.

Contrary to our expectation that a fearful face would attenuate the microsaccade rates in the rebound phase, compared to a neutral face, we did not observe a significant difference in the microsaccadic rates during the rebound phase when perceiving neutral and fearful facial expressions (Fig. 4). This finding could be attributed to the alertness of the evoked emotions. Kashihara et al. [9] used images from the International Affective Picture System [74] that could be perceived as direct threats such as snakes and guns as unpleasant stimuli. Thus, the expansion rate of the pupil diameter, which is dependent on the emotional alertness degree [57], was significantly larger in the unpleasant condition than in the neutral and pleasant conditions. By contrast, we observed no significant difference in the change in pupil diameter between the neutral and fearful expressions (Fig. 2). The arousal level of the induced emotions in our study could have been lower than that of emotional images used by Kashihara et al. [9]. If the microsaccade rebound process reflects the selective function of the importance of input visual information, then the visual information of emotional stimuli in our study may have been difficult to selectively retain because of low emotional alertness. However, whether the criteria for information selection in the microsaccade rebound process is based on emotional alertness or the degree of emotional value remains unclear. Therefore, further research is needed.

In our study, consistent with our expectation that eye gaze would facilitate microsaccadic responses opposite to the gaze, the microsaccade direction in the rebound phase (i.e., 400–600 ms relative to the onset of facial stimulus presentation) was biased to the direction opposite to the gaze, regardless of facial expression. With regard to intrinsic covert attention caused by cues presented in the visual field center that imply the direction of target appearance, the generated microsaccades are biased in the cue-indicated direction [7]. By contrast, with regard to exogenous attention automatically generated by cues presented around the visual field, microsaccades are biased in the opposite direction of the cues [15, 18]. Previous studies [7, 15, 18] have reported these microsaccade biases in the rebound phase (i.e., 200–600 ms relative to the stimulus onset of a directional cue presentation). This phenomenon can be explained by the “inhibition hypothesis” [20]. In this experiment, the participants were required to maintain a central fixation and required to suppress reflexive saccade movements due to gaze perception [27]. This saccade inhibition may have caused bias in the opposite direction.

A contradiction exists between our findings and the “IOR hypothesis” [17], which is the interpretation of microsaccade bias in opposite directions. A previous study [75] directly compared the automatic attention orientation of social cues (i.e., gaze) and nonsocial cues (i.e., peripheral cues) and reported that the social cues did not cause IOR. This finding suggests that the importance of social information negates the subcortical mechanisms underlying IOR [75]. Our findings regarding RT confirmed that attention was facilitated in the gaze direction and did not show IOR (Table 1). Nevertheless, the microsaccades were biased in the direction opposite to the gaze direction in the rebound phase (Fig. 4). These results support that microsaccades could be biased to the opposite direction of spatial attention induced by other’s gaze without IOR, which indicates that IOR may be insufficient to explain the opposite microsaccade directional bias.

Of note, our findings support that eye gazes, as well as spatial cues eliciting exogenous attention, biased the microsaccadic direction opposite to the direction of spatial attention. However, the contradiction between our results and the IOR hypothesis suggesting that microsaccadic directional bias by others’ gaze could not be fully explained by the theory on exogenous attention. We speculate that attentional orientation automatically induced by eye gaze may involve a more complex neural network for social cognition. This idea is consistent with one proposed in a previous review [29] and should be examined in the future.

With regard to the effects of facial expression on the microsaccadic directional bias, contrary to the rebound phase (i.e., 400–600 ms relative to the onset of facial stimulus presentation) in which extensive studies have reported microsaccade bias, the microsaccade direction within the interval from 800–1000 ms relative to the onset of facial stimulus presentation revealed a facial-gaze interaction effect. Moreover, inconsistent with our expectation, we observed significant microsaccadic directional bias opposite to the gaze within this phase in the neutral conditions but not in the fear condition (Fig. 5). An increase in the microsaccade rate in the same direction was also observed in the fear condition compared to the neutral condition (Fig. 5). In both "inhibition of return" and "inhibition hypothesis", microsaccade biases suppress saccades in the same direction as the spatial attentional direction induced by the cue stimulus. Thus, our results suggest that the directional bias, accompanied by the suppression of microsaccades in the same direction as eye gaze, was observed to disappear during the 800–1000 ms time period in the fear condition. Eye gaze with a fearful facial expression is a social stimulus that implies a threat toward the direction of the gaze. Shortening the duration of spatial attention toward the implied threat, rather than sustaining spatial attention, may be advantageous in survival and immediate actions (e.g., fleeing). We speculate that the disappearance of the microsaccadic directional bias for eye gaze with fearful expression in the temporally late phases will be associated with such adaptive responses in spatial attention for potential threats, which should be examined in future studies.

This finding suggested that the microsaccadic response to the gaze-mediated attention shift is differentially modulated, based on cognitive processing of the facial expression associated with the gaze. Together with the aforementioned results (post-presentation interval from 400 to 600 ms), this finding suggested that the microsaccadic response may reflect social cognitive processing. The mechanism underlying this phenomenon remains unclear; however, the microsaccadic response could reflect an individual’ s attentional traits. In this study, we conducted exploratory correlation analysis to preliminarily investigate the association between microsaccade response and an individual’s attentional trait. The results suggested that low microsaccadic responses (i.e., the microsaccade rate and microsaccade directional bias score) in the interval of 800–1000 ms relative to the onset of a gaze with fearful expression were associated with inattentive tendencies (CAARS subscale A: inattention/memory problems) (Table 3 and Fig. 6). This finding indicated that the microsaccadic response to perceiving other’s gaze with their facial expressions may reflect attentional function, possibly associated with an impairment pattern of inattention in ADHD.

Studies have indicated that patients with ADHD have deficits in oculomotor control. Previous studies have reported that, compared to typically developing individuals, individuals with ADHD have a lower ability to maintain fixation [76] and suppress inappropriate saccades [77]. Compared to neutral expressions, fearful expressions enhance attention to the gaze direction [60, 62–66]; therefore, they involve greater difficulty [27] in suppressing saccades induced by gazes with fearful expressions. Such difficulties in suppressing saccades and maintaining fixation may cause low microsaccade rates, because the microsaccade occurs during fixation [1, 2]. Additionally, the inhibition hypothesis [20] suggests that difficulties in suppressing saccades and in maintaining fixation are associated with attenuated microsaccadic directional bias opposite to the direction of the spatial attention. These findings suggested that individuals with a high ADHD tendency may be more strongly distracted to gaze cues of fearful facial expressions, and therefore may not be able to maintain fixation and may have lower microsaccade responses during fixation. With regard to the attention function in individuals with ADHD, evidence for a close relationship between attention and oculomotor control mechanisms [70] in psychological, functional anatomical, and cellular levels [78] suggests that the inattentional properties of ADHD could involve inhibitory deficits in ocular motor behavior [79]. Our findings support this concept; moreover, our study is the first to indicate that the features underlying eye movement inhibition in ADHD may be shown as a microsaccade bias toward spatial attention.

Some limitations require acknowledgment. First, we only used the fearful face of an unknown person as a social signal, which is an important social cue implying that fearful objects exist in the line of sight. Second, our findings suggesting an association of microsaccadic responses to others’ gaze with ADHD tendencies are preliminary. Additionally, the participants were limited to healthy young men without an ADHD diagnosis. In future studies, these findings should be tested by using other populations, including individuals with neurodevelopmental disorders such as ADHD.

## Conclusions

We examined the effect of others’ gaze on the observer’s microsaccades. First, we found that conscious perception of others’ gaze guided the observer’s attention and microsaccade biases in the opposite direction of the gaze. These biases showed different modulations, depending on the cognitive processing of the facial expressions of the gaze. In particular, microsaccade bias in gazes with fearful facial expressions may reflect ADHD-related characteristics. This finding suggested that microsaccade modulation by cognitive gaze processing may reflect the neural mechanisms underlying the ADHD characteristics such as inattention.

Our findings suggested that microsaccades are associated with social cognitive processing, as well as attention and emotional processing, which may contribute to the development of a theory regarding microsaccades function in social cognition. Microsaccade studies on social cognition may help elucidate the pathophysiological responses to psychiatric disorders, including ADHD. Therefore, our findings further indicate the utility of microsaccades as biomarkers for an ADHD diagnosis.

### Supplementary Information

**Additional file 1: Supplementary Figure 1. **Eye movements and microsaccade detections during fixation. **Supplementary Figure 2.** Average microsaccadic rates and the function of detection threshold λ. **Supplementary Figure 3.** Relationship between the peak velocity and the amplitude of detected microsaccades and their histograms.

## Data Availability

The datasets generated during and/or analyzed during the current study are available from the corresponding author on reasonable request.

## Abbreviations

ADHD: Attention-deficit/hyperactivity disorder
ANOVA: Analysis of variance
CAARS: Conners’ Adult ADHD Rating Scale
DSM-IV: Diagnostic and Statistical Manual of Mental Disorders, Fourth Edition
IOR: Inhibition of return
RT: Reaction time
SOA: Stimulus-onset asynchrony
