# Supplementary material for: Effects of others’ gaze and facial expression on an observer’s microsaccades and their association with ADHD tendencies
Source: J Physiol Anthropol. 2023 Sep 7;42:19. doi: 10.1186/s40101-023-00335-2 (PMC10486107; doi:10.1186/s40101-023-00335-2)
Supplement: Supplementary file 1 — Additional file 1: Supplementary Figure 1. Eye movements and microsaccade detections during fixation. Supplementary Figure 2. Average microsaccadic rates and the function of detection threshold λ. Supplementary Figure 3. Relationship between the peak velocity and the amplitude of detected microsaccades and their histograms. [file 40101_2023_335_MOESM1_ESM.docx]

**Supplementary File**

**Microsaccade detection**

Microsaccades were detected from eye movement records while the participant was staring at a stationary object. Supplementary Figure 1 shows the sample data during continuous fixation within 1200 ms (black lines) with examples of detected microsaccades (red lines) and elliptical shapes (blue lines) in the left and right eyes. Microsaccades are defined as ballistic binocular movements [60]. Therefore, to detect microsaccades, we used a detection algorithm that employs the temporal overlap criteria of binocular microsaccades in the two-dimensional velocity space [7]. Among the microsaccades separately detected in the right and left eyes, those with a temporal overlap were defined as binocular microsaccades. The specific detection procedures were as follows:

1. The viewpoint position at time (t) was converted into velocity by using the following formula:

　　$\vec{v}_{x} = \frac{\vec{x}_{n+2}+\vec{x}_{n+1}-\vec{x}_{n-1}-\vec{x}_{n-2}}{6\Delta t}$, $\vec{v}_{y} = \frac{\vec{y}_{n+2}+\vec{y}_{n+1}-\vec{y}_{n-1}-\vec{y}_{n-2}}{6\Delta t}$

This formula is based on a method developed by Engbert and Kliegl [7] in which the time series of the eye positions is transformed to velocities with the formula:

$$V\to_{n}=X\to_{n+2}+X\to_{n+1}-X\to_{n-1}-X\to_{n-2}6\Delta t,$$

which represents a moving average of velocities over five data samples to suppress noise.

1. The standard deviation, based on the median, was calculated for each velocity vector in the X and Y directions.
2. The elliptical detection threshold was set as a λ value multiplied by the calculated standard deviation (the calculation method of λ is described later).
3. Intervals continuously exceeding the detection threshold of ≥5 ms were included in the analysis to distinguish microsaccades from other data (e.g., noise from the experimental instrument).
4. Among the target intervals, those with a temporal overlap between the left and right eyes (i.e., binocular) were selected for microsaccade detection.
5. Among these, intervals within a 60-minute arc (1°) and with a maximum velocity of ≤100°/s were considered as microsaccades [53, 61].

**Determining the detection threshold parameters**

To obtain the λ value in Step 3, we set the value maximizing the signal-to-noise ratio in the detection algorithm. First, the amplitude-adjusted random shuffle of the original velocity data was conducted to only randomize the phase to create the surrogate time-series data. This process allowed the creation of data in which only the microsaccade binocularity was removed. For the created surrogate and original time-series data, we changed the λ of the algorithm; moreover, we calculated the average microsaccadic rates in all trials as λ functions. We calculated the range in which the difference was maximized between the average microsaccadic rates of the surrogate and original time-series data. The difference between the actual and surrogate data was largest with a λ value of 3–4. In this study, we set the λ value as 3. More details are shown in the Supplementary file (Supplementary Figure 2).

**Validation of the detected microsaccades**

Supplementary Figure 3 shows the peak velocity as a function of the amplitude of the detected microsaccade [3 (a)], the frequency of peak velocity occurrence [3 (b)], and the amplitude of the detected microsaccades [3(c)]. With regard to the peak velocity, the occurrence frequency had the highest increase between 40°/s and 60°/s. Moreover, the average peak velocity was 55.8°/s. With regard to amplitude, the occurrence frequency was highest at 0.133° while the average amplitude was 0.286°. These feature values are within the range reported by Martinez et al. [61]; therefore, the detected microsaccades were considered valid. Microsaccades have a ballistic nature similar to normal saccades and show a high correlation between peak velocity and amplitude [7, 53]. In this study, the detected microsaccades had a high degree of correlation (*r* = 0.69). Microsaccades and saccades have overlapping characteristics; furthermore, setting a clear threshold for distinguishing them is difficult. Therefore, based on previous studies [53, 61], we set a maximum velocity of ≤100°/s and an amplitude of ≤1° as the criteria for microsaccades (the aforementioned procedure 6).


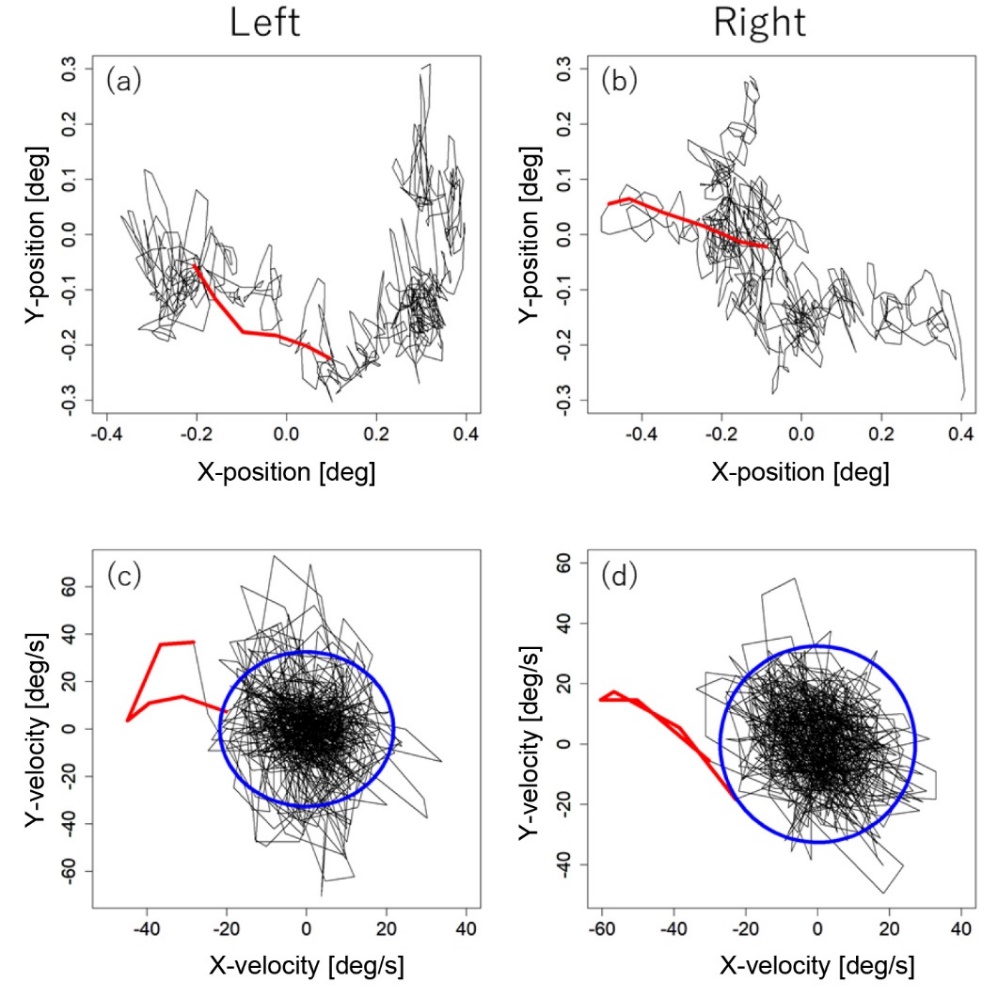


**Supplementary Figure 1. Eye movements and microsaccade detections during fixation**

Images (a) and (b) show the viewpoint trajectories (black line) recorded from the left and right eyes, respectively. Microsaccades are small and rapid events often identified by their linear appearance (red line). Images (c) and (d) show the orbital plot of eye movements recorded from the left and right eyes, respectively, in the two-dimensional velocity space. The maximum velocity of microsaccades is higher than that of other eye movement components during fixation. The detection threshold was separately calculated for the horizontal and vertical components and set in an elliptical shape (blue line).

deg: degree, s: second


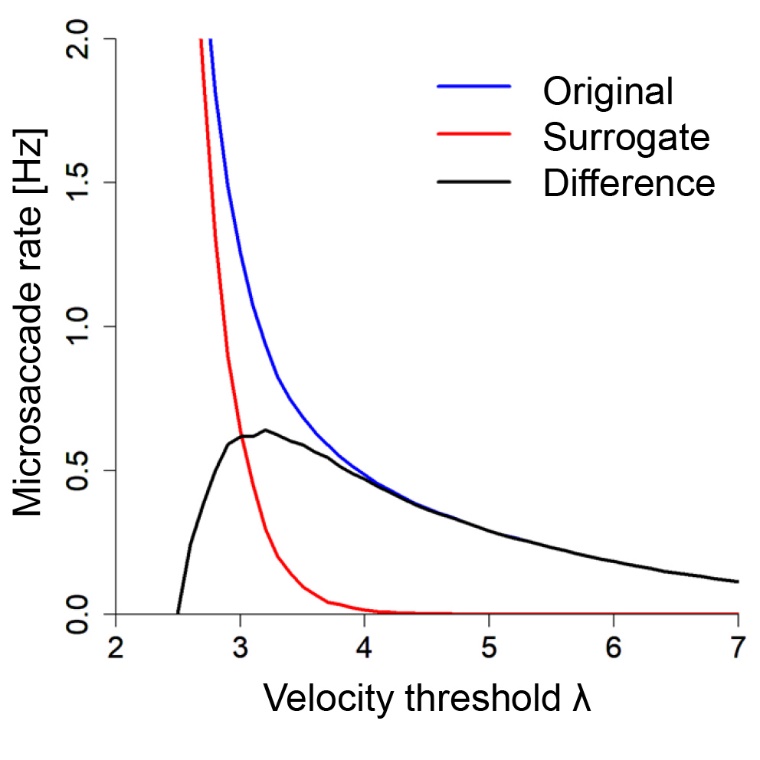


**Supplementary Figure 2. Average microsaccadic rates and the function of detection threshold λ**

The actual data (i.e., original), surrogate data, and their difference. The difference between the actual data and surrogate data is largest at a λ value of 3–4.
deg: degree, s: second


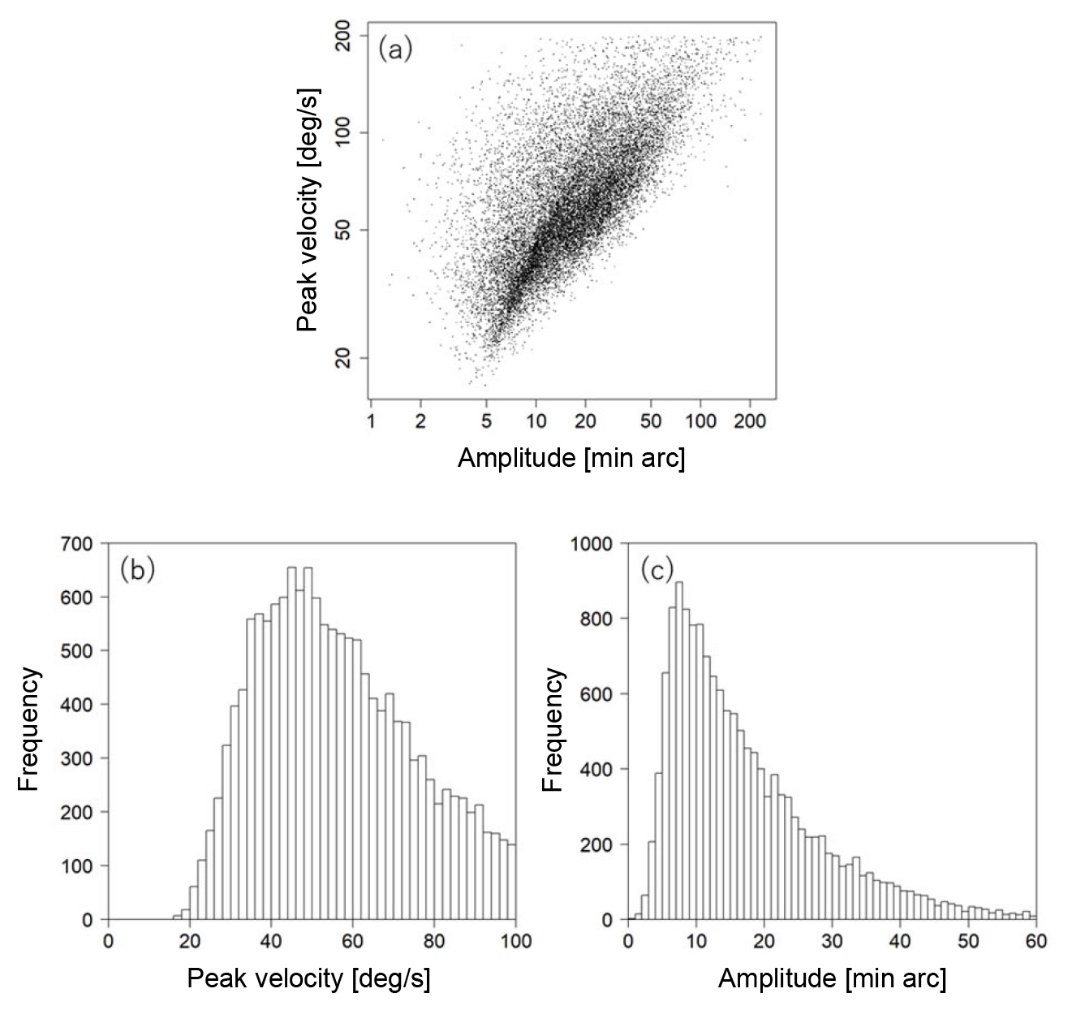


**Supplementary Figure 3. Relationship between the peak velocity and the amplitude of detected microsaccades and their histograms**

(a) The scatter plot shows a correlation between the peak velocity and amplitude. (b) The histogram shows the peak velocity occurrence of the detected microsaccades. (c) The histogram shows the frequency of the amplitude of the detected microsaccades.

deg: degree, s: second
